# Perinatal Determinants of Child Maltreatment in Japan

**DOI:** 10.3389/fped.2020.00143

**Published:** 2020-04-15

**Authors:** Haruna Kawaguchi, Takeo Fujiwara, Yoko Okamoto, Aya Isumi, Satomi Doi, Takeshi Kanagawa, Tadashi Kimura, Nobuaki Mitsuda

**Affiliations:** ^1^Department of Maternal Fetal Medicine, Osaka Women's and Children's Hospital, Osaka, Japan; ^2^Department of Global Health Promotion, Tokyo Medical and Dental University, Tokyo, Japan; ^3^Department of Obstetrics and Gynecology, Osaka University Hospital, Osaka, Japan

**Keywords:** child maltreatment, perinatal factors, the maternal and child health handbook, the child protection register, screening high-risk families during pregnancy

## Abstract

**Background:** Child maltreatment induces significant health problems, both during childhood and into adulthood. To prevent child maltreatment, it is important to detect perinatal risk factors for earlier intervention. The aim of this study was to evaluate the perinatal risk factors associated with child maltreatment during pregnancy.

**Methods:** A case-control study was conducted to compare perinatal data from the Maternal and Child Health Handbook between the case and control groups. Cases were collected from children registered in two Child Guidance Centers in Japan. The control group consisted of 3.5-year-old children in a city in Osaka Prefecture whose mothers responded to questionnaires containing information from the Maternal and Child Health Handbook. The association between perinatal factors and child maltreatment was assessed using multiple logistic regression analysis.

**Results:** The data of 70 cases and 345 controls were collected. The following were found to be perinatal factors related to child maltreatment: teenage pregnancy (OR: 257.3, 95% CI: 17.3–3832.7), a mother aged 20–24 years (OR: 22.8, 95% CI: 4.4–117.8), a father who is older than the mother by 10 years or more (OR: 14.1, 95% CI: 2.1–94.8), an unmarried mother (OR: 15.7, 95% CI: 2.6–93.6), maternal mental disorder (OR: 48.9, 95% CI: 9.3–258.3), the first maternal prenatal visit being later than 20 weeks (OR: 132, 95% CI: 12.7–1384.7), little prenatal care (<10 visits) (OR: 21.4, 95% CI: 2.9–157.1), a low-birth-weight baby (OR: 5.1, 95% CI: 1.1–24.1), and congenital disease (OR: 7.9, 95% CI: 1.1–56.4).

**Conclusions:** This study revealed that young mothers, fathers much older than mothers, unmarried mothers, and maternal mental disorder, mothers with late first visit or little perinatal care, and low-birth-weight babies and babies with congenital disease were associated with child maltreatment. These findings can be used to detect high-risk families for child maltreatment during or after pregnancy.

## Introduction

Child maltreatment causes significant problems in child development (1–3). It is a serious global problem and Japan is no exception (4). The latest data indicates that, from April 2016 to March 2017, a total of 49 deaths were due to child abuse (5). Among the deaths, children under 1 year of age accounted for 32 (65.3%), of which 16 (50.0%) were within the first month of life. These numbers suggest the importance of early prevention efforts against child maltreatment from pregnancy onward.

Maternal perinatal risk factors for child maltreatment have been reported and the recognition of families with a high risk of child maltreatment through gestation is an effective means for early prevention (6–9). This is reasonable approach for early detection of child maltreatment, which uses data that are easily measured during regular prenatal care. Therefore, in this survey, we used the Maternal and Child Health Handbook (MCH). In Japan, a woman is issued with the MCH after pregnancy is confirmed (10, 11). Japan has a pregnancy registration system, subsidies for pregnancy health checkups, and a universal healthcare system. The MCH is a comprehensive home-based health booklet for mothers and children that is distributed at local government offices. It records the health condition of the mother throughout pregnancy, delivery, and the postnatal period, as well as the condition of the child's development and immunization. In addition, it plays a role in health education about pregnancy, birth, neonates, childcare, nutrition, dental care, and immunization. Medical records are written down in the handbook by obstetricians, pediatricians, midwives, and public health nurses at hospitals, clinics, or health centers. Parents bring the handbook to hospitals or clinics when their children receive a routine vaccination or fall sick. Pregnant women receive regular (at least 14) prenatal checkups at obstetric facilities. In a prenatal checkup, the mother's health condition is assessed and a medical examination and health guidance are conducted. A newborn receives a medical examination at an obstetric facility at one month old with the mother, and subsequently, an infant medical examination is conducted in municipal health centers at the age of 3 months, 1.5 years, and 3.5 years (12, 13). In Japan, the reason for placing children on the child protection register is not only child maltreatment but insufficient nurturing, such as when their mother has a disease. We examined the perinatal risk factors related with child maltreatment listed in the MCH, by comparing children who had been maltreated and registered at a Child Guidance Center with children who had never been maltreated up until their 3.5-year health examination.

## Materials and Methods

This was an unmatched case-control study comparing perinatal data from the MCH between children in foster care and those who had never been registered for child maltreatment.

In this study, the cases comprised 0- to 5-year-old children who had been placed on the child protection register for child maltreatment at two Child Guidance Centers in Osaka Prefecture. The cases were selected from the entire area of Osaka Prefecture and from the surrounding prefecture. We limited the sample to mothers with their copies of the MCH. We asked health nurses on the staff of the two Child Guidance Centers to provide information about the children placed on the child protection register. We received information regarding their MCH from the nurses with the exception of personal information such as name, address, and birth date. We could not obtain the consent from parents of the cases because the cases had been placed on the child protection register for child maltreatment. Instead, the cases survey was conducted with the consent of the Child Guidance Centers and Osaka Prefecture. The control group consisted of 3.5-year-old children living in a city in Osaka Prefecture whose mothers had agreed to be the subjects of this study at the infant health examination. We obtained the written informed consent from the parents of participants. The mothers answered a questionnaire, including information regarding the MCH. On the questionnaire a total of 27 question items were set, and closed or multiple-choice question was prepared for the respective items with the exception of maternal and paternal age, gestational age, the week of the first visit, number of pregnancy medical examinations and birth weight. The public health nurses in the city confirmed the information given in the questionnaires, and if there were any blank responses, they asked the mothers to complete them. This questionnaire survey was conducted among all mothers who have 3.5-year-old children living in the city. As a result, the survey was also conducted with mothers of children requiring the city's care because of maltreatment. In Japan, the most severely affected children are placed on the child protection register and less severely affected ones are living with their parents and are watched under the city's care. However we excluded them from the control group. In addition, we also excluded children who had no detailed information in their MCH. The sample size was calculated using the assumption of 95% confidence interval, and 80% power with one to five ratio of cases and controls using the hypothetical proportion of 5% with perinatal risk in controls, to detect an odds ratio of 3.0 or greater.

We examined the following perinatal factors: background factors, gestational factors, and children's factors. The background factors consisted of maternal age, paternal age, a much older father, number of children (including the subjects of this study), economic status, partner status, and maternal mental disorder. Economic status was defined as whether welfare or public assistance benefits had been received for the cost of delivery. Among the cases, maternal mental disorder included mothers who were considered to have psychiatric problems by the foster home staff, as well as those who were diagnosed with a mental disorder. In the control group, information about maternal mental disorder was composed of self-reported data from the questionnaire. In the questionnaire, it was included as one of the options to obtain the medical history. The gestational factors consisted of the week of the first visit, number of pregnancy medical examinations, hypertensive disorder of pregnancy, proteinuria, blood transfusion, and method of delivery, which were obtained from the MCH. The children's factors consisted of multiple pregnancy, congenital disease of children, birth weight, and gestational age at delivery.

We compared continuous variables using the Wilcoxon rank-sum test because the variables did not have a normal distribution, and categorical variables using Pearson's chi-square and Fisher's exact tests. The association between maternal mental disorder or maternal age and other factors was examined by chi-square test with cross tabulation. Perinatal factors related to child maltreatment were evaluated using multiple logistic regression analysis after adjusting the age of admission to the child protection register or the age at the time of investigation. The Variance Inflation Factor (VIF) was calculated using the regression analysis to confirm multi-collinearity in the multivariate analysis. There were some significant variables in the univariate analysis that were excluded in the multivariate analysis. Twin pregnancy was not included in the multivariate analysis because there were only 10 pairs across both groups. Because proteinuria and hypertensive disorder of pregnancy, maternal and paternal age, and premature birth and cesarean section are closely related, we selected only hypertensive disorder of pregnancy, maternal age, and preterm birth as factors, respectively, for the multivariate analysis. The week of the first visit and number of pregnancy medical examinations were correlated. Therefore, the factor of the week of the first visit being before 20 weeks' gestation and with more than 10 examinations was used as a reference for two situations: the first visit being after 20 weeks' gestation, regardless of the number of examinations, and the first visit being before 20 weeks' gestation with less than 10 examinations. Statistical analyses were performed using Stata software, version 14.0 (Stata). Two-sided *p*-values of less than 0.05 were considered to indicate statistical significance.

## Results

**Figure 1:**
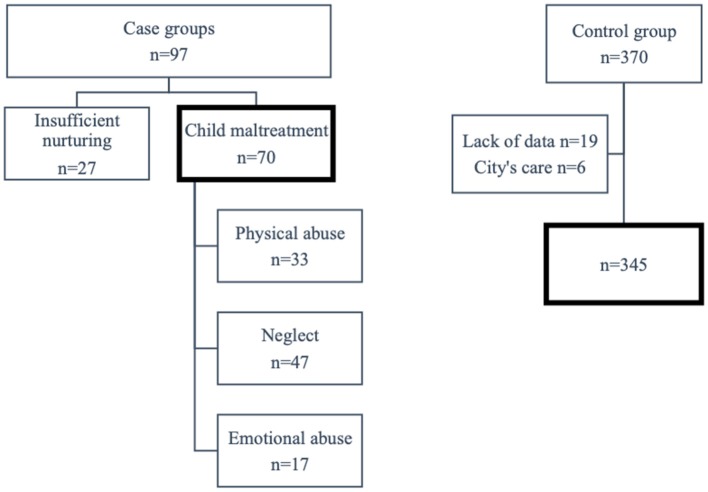
The flowchart of study population. A total of 27 children were excluded because they had been placed on the child protection register because of insufficient nurturing, not child maltreatment. Among the causes, physical abuse accounted for 33 (47.1%); neglect, 47 (67.1%); and emotional abuse, 17 (24.3%). The type of abuse was duplicated in some cases. A total of 25 cases were excluded either because of lack of detailed information (*n* = 19) or because of children requiring aid due to maltreatment (*n* = 6).

Between April 2013 and March 2016, there were 97 children aged 0 to 5 years who had been placed on the child protection register at the two Child Guidance Centers in Osaka Prefecture and who had their copies of the MCH. A total of 27 children were excluded because they had been placed on the child protection register because of insufficient nurturing, not child maltreatment. The 70 remaining children had been placed on the child protection register because of child maltreatment. Among the causes, physical abuse accounted for 33 (47.1%); neglect, 47 (67.1%); and emotional abuse, 17 (24.3%). The type of abuse was duplicated in some cases (Figure 1).

From May 2015 to April 2016, 576 children aged 3.5 years underwent the infant health examination at a city in Osaka Prefecture. There were 370 (64.2%) of their mothers who agreed and responded to the survey. A total of 25 cases were excluded either because of lack of detailed information (*n* = 19) or because of children requiring aid due to maltreatment (*n* = 6). Thus, 345 cases were included in the analysis. Table 1 shows the comparison of background factors, gestational factors, and children's factors in the registered and control groups. There was a significant difference between the registered and control groups in almost all the factors. The proportion of young parents was significantly higher in the cases than in the control group. In addition, cases in which the father was more than 10 years older than the mother comprised 20.7% of the registered group, which was significantly higher than in the control group (5.3%). The proportion of unmarried parents among the cases was 50%, which was significantly higher compared to the control group (2.9%), and 82.3% of those were teenagers. Further, 48.5% of the cases had received financial assistance for helping with birth costs, which was significantly higher compared to the control group (8.2%; *p* < 0.001). Among the cases there were 33(47.1%) mothers with mental disorder, 26 of whom (78.8%) were diagnosed by a doctor. This rate was significantly higher than that in the control group (3.8%). A statistically significant difference was seen between the groups with regard to gestational age at the first visit and number of pregnancy medical examinations. Among the cases, five mothers had delivered a baby without a pregnancy examination. Hypertensive disorder of pregnancy, proteinuria, cesarean section, and preterm birth were all significantly higher in the cases than in the control group. The number of preterm births and low-birth-weight infants was significantly higher in the cases than in the control group. The rate of congenital disease among children was 11.6% among the cases, which was significantly higher than that in the control group (2.9%). All the twins among the cases were preterm births, whereas all the twins in the control group were term births.

**Table 1:** Comparison of background factors, gestational factors, and children's factors in the case and control groups.

**Risk factors**	**Case group (*n* = 70)**	**Control group (*n* = 345)**	***P***
**BACKGROUND FACTORS**			
Maternal age	23 (14–40)	31 (17–43)	<0.001
Teenage pregnancy	14/70 (20.0%)	3/345 (0.9%)	<0.001
Paternal age	29 (14–72)	33 (19–54)	0.002
Father over 10 years older than the mother	12/58 (20.7%)	18/338 (5.3%)	<0.001
Number of children ≥3	29/70 (41.4%)	69/345 (20%)	<0.001
Economic poverty	33/68 (48.5%)	28/343 (8.2%)	<0.001
Unmarried mother	35/70 (50%)	10/345 (2.9%)	<0.001
Maternal mental disorder	33/70 (47.1%)	13/345 (3.8%)	<0.001
**GESTATIONAL FACTORS**			
Gestational age at the first visit	14 (7–40)	9 (4–38)	<0.001
First visit ≥20 wks^a^	16/66 (24.2%)	2/324 (0.6%)	<0.001
Number of pregnancy medical examinations	10 (0–15)	13 (7–20)	<0.001
First visit <20 wks^a^ and number of examinations <10	17/66 (25.8%)	8/319 (2.5%)	<0.001
Hypertensive disorder of pregnancy	8/66 (12.1%)	5/341 (1.5%)	<0.001
Proteinuria	44/66 (66.7%)	119/341 (34.9%)	<0.001
Blood transfusion	1/54 (1.9%)	4/343 (1.2%)	0.67
Cesarean section	29/70 (41.4%)	56/343 (16.3%)	<0.001
Gestational age of delivery	38 (28–41)	39 (33–42)	<0.001
Preterm birth	18/69 (26.1%)	11/345 (3.2%)	<0.001
**CHILDREN'S FACTORS**			
Twins	6/70 (8.6%)	10/345 (2.9%)	0.025
Children's age at registration or investigation	2.5 (0–5)	3.5	
Congenital disease	8/69 (11.6%)	10/345 (2.9%)	0.001
Birth weight (gms)	2723 (828–4002)	3034 (1222–4182)	<0.001
Low birth weight	23/70 (32.9%)	28/343 (8.2%)	<0.001

a *wks - weeks' gestation*.

*There was a significant difference in the registered and control groups in almost all of the factors. Data are given as median (range) or n (%)*.

The results of the multivariable logistic regression analysis for perinatal factors associated with child maltreatment are presented in Table 2. The analysis was adjusted for the age of admission to the child protection register or the age at the time of investigation. In cross tabulation, maternal mental disorder was associated with younger mother, father over 10 years older than the mother, an unmarried mother, poverty, the first maternal prenatal visit being later than 20 weeks, little prenatal care, prolificacy, hypertensive disorder of pregnancy, preterm birth, and low-birth-weight baby. Moreover, teenager was associated with father over 10 years older than the mother, an unmarried mother, poverty, the first maternal prenatal visit being later than 20 weeks, little prenatal care, and prolificacy. However, the VIF of the final model was 1.29, which was small enough to conclude this analysis was still valid. As perinatal factors related to child maltreatment, we observed the following: teenage pregnancy (OR; 257.3, 95% CI;17.3 to 3832.7), a mother aged 20–24 years (OR; 22.8, 95% CI;4.4 to 117.8), a father older by 10 years or more than the mother (OR;14.1, 95% CI;2.1 to 94.8), an unmarried mother (OR;15.7, 95% CI;2.6 to 93.6), maternal mental disorder (OR;48.9, 95% CI;9.3 to 258.3), the first maternal prenatal visit being later than 20 weeks (OR;132, 95% CI;12.7 to 1384.7), little prenatal care (less than 10 visits) (OR;21.4, 95% CI;2.9 to 157.1), a low-birth-weight baby (OR;5.1, 95% CI;1.1 to 24.1), and congenital disease (OR;7.9, 95% CI;1.1 to 56.4). The area under receiver operating characteristic curve (AUC) was 0.971 (95% CI = 0.942 to 1.000).

**Table 2:** Multivariable analyses for perinatal factors associated with children in foster care compared with non-abused children.

**Risk factors**	**cOR^b^ (95% confidence interval)**	**aOR^c^ (95% confidence interval)**
Maternal age	0.8 (0.8 to 0.9)	
<20	45.1 (12.3 to 165.1)	257.3 (17.3 to 3832.7)
20–24	9.7 (4.9 to 19.1)	22.8 (4.4 to 117.8)
>25	1.0	1.0
Parents' age gap (father's – mother's)		
<10	1.0	1.0
≥10	4.6 (2.1 to 10.3)	14.1 (2.1 to 94.8)
Paternal age	0.9 (0.88 to 0.97)	
Single		
Yes	33.5 (15.3 to 73.4)	15.7 (2.6 to 93.6)
No	1.0	1.0
Number of children		
1	2.2 (1.1 to 4.3)	0.2 (0.04 to 1.3)
2	1.0	1.0
3	2.8 (1.4 to 5.6)	1.0 (0.2 to 4.9)
≥4	10.1 (3.8 to 36.6)	9.3 (0.9 to 93.1)
Economic poverty		
Yes	10.6 (5.7 to 19.6)	3.6 (0.8 to 16.8)
No	1.0	1.0
Maternal mental disorder		
Yes	22.8 (11.0 to 47.1)	48.9 (9.3 to 258.3)
No	1.0	1.0
Gestational age at the first visit^d^	1.4 (1.3 to 1.5)	
Number of pregnancy medical examinations	0.5 (0.4 to 0.6)	
First visit week ≥20 wks^a^	89.0 (19.8 to 398.9)	132 (12.7 to 1384.7)
First visit week <20 wks^a^ and number of examinations <10	19.9 (8.0 to 49.6)	21.4 (2.9 to 157.1)
First visit week <20 wks^a^ and number of examinations ≥10	1.0	1.0
Missing data	0.4 (0.1 to 2.7)	
Hypertensive disorder of pregnancy		
Yes	9.3 (2.9 to 29.3)	2.9 (0.3 to 30.8)
No	1.0	1.0
Proteinuria^d^		
Yes	3.7 (2.1 to 6.5)	
No	1.0	
Blood transfusion		
Yes	1.6 (0.2 to 14.6)	
No	1.0	
Cesarean section		
Yes	3.6 (2.1 to 6.3)	
No	1.0	
Gestational age of delivery	0.7 (0.6 to 0.8)	
Preterm birth		
Yes	10.6 (4.7 to 23.7)	1.52 (0.2 to 15.2)
No	1.0	1.0
Twins		
Yes	3.1 (1.1 to 8.9)	
No	1.0	
Birth weight (gms)	0.99 (0.99 to 0.99)	
Low-birth-weight baby		
Yes	5.5 (2.9 to 10.3)	5.1 (1.1 to 24.1)
No	1.0	1.0
Congenital disease		
Yes	4.4 (1.7 to 11.6)	7.9 (1.1 to 56.4)
No	1.0	1.0

a wks - weeks' gestation;

b crude odds ratio;

c *aOR - adjusted odds ratio*.

*The values were adjusted for the age of admission to the child protection register or age at the time of investigation*.

## Discussion

We investigated the perinatal factors from the MCH associated with child maltreatment, by comparing children who had entered foster care with those who had never been registered for child maltreatment. The background factors found to be related to child maltreatment were being a mother aged between 20 and 24 years, teenage pregnancy, a father over 10 years older than the mother, an unmarried mother, and maternal mental disorder. However, age differences between parents are associated with whether it is the first marriage or a remarriage. Thus, it is uncertain in this study if older fathers were an independent factor as we did not collect data about the number of marriages.

In this study the maternal mental disorder was particularly strongly associated with child maltreatment. The inspection of the child abuse death in Ministry of Health, Labor and Welfare of Japan also confirmed that the maternal mental disorder was a contributing factor in the child abuse death (5). The maternal mental disorder was also related to other background factors and gestational factors. The presence of a mental disorder was associated with poverty and a deterioration of cognitive function, which may lead to little prenatal care and hypertension due to poor self-health management. In addition, preterm birth or low-birth-weight baby can increase the burden of childcare, which may lead to deteriorate the mother's mental status in the postpartum period.

The gestational factor related to child maltreatment was mothers with a late first visit or little prenatal care. In Japan, prenatal checkups at obstetric facilities are scheduled frequently (at least 14 times). The median number of prenatal checkups in the control group was thirteen, which was significantly higher than ten among the cases. Ten visits may be adequate in some countries; however, skipping scheduled medical checkups for any reason may be presumed to be related to child maltreatment. Moreover, low-birth-weight babies and babies with congenital disease were associated with a significant increase in the odds of subsequent child maltreatment, which was consistent with previous studies (14–16). Risk factors of child maltreatment and perinatal records have been reported to be related. Kelly et al. found an association between perinatal records and subsequent abusive head trauma; the risk factors of abusive head trauma were being young, single, and Maori; having shorter interpregnancy intervals, rupture of membranes for longer than 48 h, and an early gestational age at delivery; and engaging in formula feeding in the first week of life (17). Further, a large UK cohort study, the Avon Longitudinal Study of Parents and Children (ALSPAC), reported the risk factors during pregnancy associated with child maltreatment (7, 18, 19). In their analysis, young parents, low educational achievement, past psychiatric history, a history of childhood abuse, deprivation, and poor social networks were all significantly higher at investigation or registration in the foster home. Moreover, single parents, broken and reordered families, and low-birth-weight children were all at high risk of registration. The factors that were consistent with those in our study were young parents, past psychiatric history, and single parents. Although the status of pregnancy medical examinations was not considered in the ALSPAC study, we found that a late first and few prenatal visits were significantly related to child maltreatment.

Several instruments have been developed for the early identification of families at risk for child maltreatment and their validity has been assessed; for example, the California Family Risk Assessment (CFRA) in the USA or the Instrument for Identification of Parents at Risk for Child Abuse and Neglect (IPARAN) in the Netherlands (20, 21). While there are no such comprehensive national instruments in Japan, some obstetrics hospitals and health organizations evaluate perinatal risk using their own methods.

The strength of this study was that the factors found in this research can be readily adapted throughout Japan as the data were gathered from the MCH, which is standardized in Japan. The MCH was used in some countries other than Japan, and the information described in MCH was easily available and generally versatile.

The following limitations should be considered. First, the subjects in cases were limited to those with their copies of the MCH. The reasons for not having their copies included: not received, lost, or forgotten the MCH. Cases without the MCH may have further problems during pregnancy. Prospective research covering every registered child is required to re-evaluate our results adequately. Second, only the most severely affected children who entered foster homes were included among the cases. A different outcome might be obtained if we investigate children who required the city's care but were not severely affected enough to enter the foster home. Third, despite excluding cases of children in the city's care, undetected child maltreatment cases may have been included in the control group. Fourth, there was selection bias in the control group as the data were obtained from voluntary questionnaires and mothers' self-report. Fifth, we only examined information contained within the MCH at the infant health examination. Thus, this study did not cover some factors that have been reported to be associated with child maltreatment, such as the parent's own childhood abuse, educational qualification, and employment status. Finally, the age ranges differed between the groups; however, the analysis was adjusted for the age of admission to the child protection register or the age at the time of investigation.

After a more extensive investigation across Japan, we aspire to start screening for risk of child maltreatment during pregnancy using these factors and provide education and resources based on risk. Targeted support during pregnancy may prevent the onset of subsequent child maltreatment. We are now starting same research in several region and web research in Japan to use a simpler approach to assess the risk of child maltreatment during pregnancy.

In conclusion, this study showed some of the gestational factors (i.e., mothers with late first visit or little prenatal care), children's factors (i.e., low-birth-weight babies and babies with congenital disease), and background factors (i.e., mother under 25 years old, father over 10 years older than the mother, unmarried mother, maternal mental disorder) were strongly associated with child maltreatment. It would be important to detect mothers and children in need of support throughout the course of pregnancy and the postpartum period.

## Data Availability Statement

Data availability access is restricted by the ethics committee. Requests to access the datasets should be directed to HK, haruna@wch.opho.jp.

## Ethics Statement

This research was approved by the Osaka Women's and Children's Hospital's ethical review board (approval number 887, date of approval: March 8th, 2016). Written informed consent from the participants' legal guardian/next of kin was not required to participate in this study in accordance with the national legislation and the institutional requirements.

## Author Contributions

HK made the concept of the study design and analyzed and interpreted the patient data regarding prenatal data, and was a major contributor in writing the manuscript. TF, AI, and SD analyzed and interpreted the patient data. YO, NM, and TKa made the concept of the study design. TKi supervised this work. All authors read and approved the final manuscript.

### Conflict of Interest

The authors declare that the research was conducted in the absence of any commercial or financial relationships that could be construed as a potential conflict of interest.

## Abbreviations

MCH: the Maternal and Child Health Handbook.
